# Supplementary material for: Acceptance and compliance with micronutrient powder (MNP) among children aged 6–23 months in northern Nigeria
Source: PLOS Glob Public Health. 2022 Oct 17;2(10):e0000961. doi: 10.1371/journal.pgph.0000961 (PMC10022258; doi:10.1371/journal.pgph.0000961)
Supplement: S1 Dataset — (ZIP) [file pgph.0000961.s008.zip › S8_Dataset/In-depth Interviews.zip!/In-depth Interviews/030715_K_BK_AB_2_CG.pdf]

PHASE TWO (2) HOME-FEEDING TRIAL IN-DEPTH INTERVIEW WITH A MALE CAREGIVER AT YAMAMA KETARE COMMUNITY, BIRNIN KEBBI LOCAL GOVERNMENT AREA OF KEBBI STATE

ON 1st JULY, 2015

I: please I want you to describe people in your immediate family living in your house hold?

R: yes, we don't have any problem, they are five in number my wife two female kids and two male children, 11 years, 6years, 4 years, under 2years respectively, they don't have anything doing to look for money to help in taking care of family but, the biggest sister among them use to go out with her colleague for planting of seed during rainy season.

I: We have heard of food challenges for young children in this setting related to the seasons. What mechanism could be adopted in coping these food challenges?

R: we don't have much problem pertaining feeding o children because no matter how you most look for what children is going to eat or otherwise they won't aloud you to seep meaning that they will crying the whole night.

I. uh

R. The food we gave them are: *towo, shinkefa, kunu*.

I: I mean, we have heard of food challenges from your community here recently, we discovered that some foods are seasonal?

R: During dry season we water melon, tomatoes, spinach, amaranths, morenga, pepper, sugar cane, onion, potato, cassava, mango and even dry season rice. During rainy season we cultivated millet, sorghum, rice, maize, cowpea that is beans, ground nut, etc.

I: What mechanisms could be adopted in resist these challenges?

R: Mechanisms to cope the challenges is that we try as much as we could to store the harvested produce in order to available all year round, for example now tomatoes, pepper and onion are fresh able crops that can spoil easily, in our own case we sundry them and they are always available. So we don't have much problem with food related challenges. The ones we

don't have them here. When we need them, we go to the market to buy them. They are available in the market.

I: One thing we heard in other interviews that caregivers often give their children 6-23 months is "tea". Could you please explain "tea" for me?

R: Yes. We use buy tea in that small market close to my house is just a liquid or drinks prepared with different ingredients. He boil water in the evening time and in the morning time, during boiling he combine lipton and sugar together with water and also a small quantity of ginger, if you come to buy the tea they put is just milk and sometimes bounvita if you ask him to put it for you.

I: Thank you for that explanation. Now I want us to talk about the MNP that you received. Could you tell me about your experience with the product?

R: Truly we thank almighty Allah that he gave us the opportunity to be part of the people you selected for MNP product usage for our children. It is good for every child to be given the MNP product because it is very good for their health, making the child very active, he is now stronger and healthier, he eat well, you will see them always fresh. So my experience of MNP product to my child is good and I have seen a lot of improvement as well good changes in the health of my child.

I: OK. I want you to talk more about the positive aspects of the experience with MNP.

R: As I told you initially that he is now stronger and healthier and also good looking. If see my child the way he is playing, running up and down you get to know that he is now stronger and healthier but before the child look inactive.

I: Please sir, I want you to tell me a specific story relating to improvement in your child's health compared to before using the MNP.

R: We were all happy now our child is getting stronger, healthier and also good looking. If see the way he is playing, running up and down you get to know that he is now stronger and healthier but, before the child looking inactive, he don’t even take *kunu* but, now he take too much *kunu* and some time eat food with his mother so that is why i said, i sow so many changes on him.

I: Thanks you for your MNP story you gave to me. Could you talk about any general challenges you had during the MNP trial?

R: Initially my wife don't have the intention of given that MNP product to our child but when i come back from the city *Mai Anguwa* send someone to call me he told me everything concerning the product and how to use the product appropriately, so one to the house and told her that the product is good for our child and how to make use of MNP properly.

Honestly, if there is any challenge, I am yet to notice any.

I: Tell me about any specific barriers that made it hard to use the MNP appropriately

R: Honestly, there is no barrier.

I: Ok. Some caregivers mentioned that it was hard to store the MNP without it being taken by others or eaten by rodents. Could you please talk about your experience?

R: In our case we don't have any problem with our storage, she use to put it on box where i kept my books nothing can enter in to that box not even rodent talk less of other people in the house, in fact the you gave to us is highly protected.

I: Do you observed any side effect of the MNP on your child?

R: We did not observe any side effect on our child.

I: Some mothers indicated that it might be hard to remember to use MNP. Did you have a similar experience?

R: No we don't have any problem like that because even if she forget to gave him the MNP product in the morning when she remembered in the afternoon or in the evening she will gave him. I normally use to ask her in order to remind her that if she did not forget to gave the MNP because we are human being, we do normally forget thing some time but in the case of MNP I know she is really trying, because since the date she started administering the MNP we never forget to gave him the NMP.

I: Thanks for the information. What specific solutions could you suggest to address each of the barriers or challenges mentioned above?

R: Initially I told you that we did not encounter any problem with the MNP Product.

I: Thanks you, Let us now talk about the child's feeding and response to the MNP. Please talk about how you feed the MNP to the child usually.

R: Truly we accepted your product 100% and we are fully committed I my wife and our baby. We thank almighty Allah that gives us the opportunity to use the MNP product. Some of our people are right now saying that "how do I gate the MNP product because they too want to gate it" in fact I know some of our people are disturbed because they don't have the MNP product. What I use to said that it's just a gift from Allah so I thank Allah that gave you the opportunity to select me among the people who received the MNP product.

I: So what food do you use to mix with MNP?

R: We do mixed the MNP product with *fura, shinkafa, faten wake and kunu*.

I: When do you usually give the MNP to the child?

R: We don't have specific time to give MNP to our baby but, mostly in the morning sometime is between 11-12 noon and sometimes he may not finish till in the evening time that is after *asari* prayer before *magrib* prayer.

I: To what extent does the child likes or dislikes the taste of the MNP?

R: the baby like it very much because mostly we use *kunun koko* when mixing the product, and then he loves *kunun koko* very well, in the sense that once it is the time for us to give him that *kunun koko* he will notify us by carrying his cup the flax we use to his *kunun koko*, or by crying, whatever you gave to him he will not eat it for you until you gave him his *kunun koko*, so that is why i said he likes the MNP product very much because we mix the MNP mostly *kunun koko*.

I: Ok. I want to know how, if at all, consumption of MNP impacted the child's diet.

R: The baby food in takes have increased, compared with before he started consuming the MNP product, he reduce suckling the breast milk of his mother, once he take in his *kunun koko* that is enough for him to take time before consuming anything again.

I: What about her breast milk consumption?

R: The reduce suckling the breast milk of his mother, once he take in his *kunun koko* that is enough for him to take time before consuming anything again. The baby is very active now and I so too many change on that boy that is positive changes.

I: What about the varieties of foods consumed?

R: My baby is just around 6-7 months of age, we do normally not aloud child at his age to eat varieties of but he eats all types of foods we give to him. If you look at the child now you will see that he is becoming fresh, active, stronger and healthy but, before he is looking doll, that is in active and he is very slow in whatever he is doing that is learning. Finally we really appreciate this MNP product because it helps us in introducing our child to different kind of food that helps the child to grow bigger, stronger and healthier.

I: In other communities, we noticed that sharing of the MNP occurred. Could you talk about any sharing of the MNP when you used it?

R: No we never give it to any one apart from child that who's MNP is given for and we never jump any day since the date we started given the MNP to our baby and every day we gave him one sachet of MNP.

I: Please could you tell more on any sharing of the MNP due to food shortage during lean season?

R: Honestly, I have never heard any problem of food shortage in my house but, sometimes due to the negligence of women, if she did not prepare it she ask *kunu* from her friend so that she can mix the MNP with in order to feed the child.

I: Please tell me whether the mother or father do ate out of the MNP as a snack

R: No, by all indication she doesn't ate it. She gave it to the child alone as prescribed one daily.

I: What happens to the leftovers of the MNP?

R: We don't give him more than what he can’t finish. His mother told me that there are always no leftovers and I notice it myself. I initially told you that his feed intake increases seriously so even if you gave him what you know he can finish, he will still ask for more.

I: Tell me whether 60 sachets will be enough for 60 days.

R: Yes, we need it even if it’s more than 6o sachet.

I: Why did you say they will not be enough?

R: B because you might loos one or two do to our negligence so we need replacement and there is no any additional one to replace, even 60 will be enough but, we are urging if you have any chance of adding another sachets above 60 sachets do as much as possible.

I: Ok. Now that you are using the MNP, could you talk about how you perceive it?

R: My perception is that the MNP Powder is very easy to use. Just as the person who brought it explained, he said that it can be administered in the morning, afternoon and evening. But he recommended morning. So that, when one forgets to administer it in the morning, he/she can remember before the day runs out.

I: Ok. Do you see the MNP as food, medicine or something else?

R: Honestly I see it as medicine.

I: OK. Why?

R: Because the way you prescribe it to me is almost the same way that the health personnel in the clinic use to prescribe the medicines for us when we are seek and because it helps in improving the health status of the children's.

I: If this product were cheaply on sale in your community, would you purchase it?

R: Yes, I can buy, because it seems to be good for my child, it's making him stronger and healthier. So I am ever ready to buy whatever is good and improvement for his health.

I: How much would you be willing to pay for a sachet?

R: Yes, I can buy it at the rate of 100 naira per sachet. Because I sow the product positive effect on child meaning that I where we come from with baby and I know where we are right now so, the boy is doing the great. No matter how much you fix on the product, to me is not costly because if you calculate the total amount of money I will spend when he is seek, it will higher than the amount of MNP product.

I: You are giving me great information. Now please tell me some ways that you believe would be effective for promoting MNP to caregivers in the community so that they would comply with using it appropriately

R: Honestly, when you want promote the MNP product is by bringing enough of you MNP product, so that our people will have it for their children, enlightenment campaigns, educating our people on how the MNP product should be given; how it should be use; its importance and positive effect on the children. If possible use our indigenous people to be explaining these product and use of female for their female counter part. If that can be achieved I assured you that the product will reach where you are not even expecting.

I: Are there other ways we can follow to ensure the caregivers use it appropriately?

R: You can only achieve appropriate use of the product by the use of husband or any influential person the house, so that he will be asking and reminding them if the product is been use appropriately or not.

I: Thank you so much on that information. What people most influence your opinions and knowledge about feeding practices?

R: In any community they have their own settings and in any religion they have their own settings, I am a muslim and then you know, we have our own way of feeding behaviour that is we are expected to do, for example the mother or the father are the one feed the child of

under two years or the elder sisters or brothers too can feed the chid. So my religion is the one that influence opinion the feeding practice.

I: Ok. Who influenced your decisions regarding feeding your baby with the MNP?

R: You know I told you that I appreciate the way our village head is handing issues like that because treated everybody equally. He send somebody to call me in my house I mate him discussing MNP issues with person in charge of MNP distribution that is Malam Altine, they explain everything to me pertaining MNP usage and advantages.

I: Where would be good places to disseminate health information related to MNP?

R: The good place to disseminate health information related to MNP is in our village head palace or the mosque on Friday because is only easiest way to gather people of this community.

I: Thank you for that explanation. Who in your community do you trust the most for receiving messages about infant and young child feeding?

R: The person I can trust in my community is our village head and our Imam, because ones we have any problems they are the ones that we will run to, whatever concern this village they worked hand in hand to find the best solution to us and our community.

I: What forms of media do you prefer for receiving MNP messages? Brochure/Flyer/Posters?

R: Yes, i know posters are very good information displayer for any king of information you want to send to us because majority of us cannot read or write but we all understand posters.

I. uh uh

R. because is something that you can see and sometimes you can get detail information about the MNP.

I: What about word-of-mouth?

R: Word-of-mouth is not always good method of sending or receiving MNP message because for to gather people is not easy and is not possible to meet with us one by one due to it's time consuming and hard to meet every one in the community here.

I: What about radio?

R: Radio too is good but is not everyone that will attach seriousness to it, because we don't even have much time to attach to the radio more especially during rainy season, so even when a serious message is passed, we can't take it as serious as that of posters or word-of-mouth.

I: What about SMS?

R: For the SMS only once that have the hand set or phone can receive SMS and even those that have the phone can read the message and understand the MNP programme.

I: What other ways?

R: The once we mention earlier is enough to receive or send MNP message.

I: Could you please tell me what ways would you prefer to receive education regarding IYCF and MNP?

R: I initially told you that it should be through the health personnel in this community and though our village head and our religious leader and other influential people in this community.

I: Thanks so much for that suggestions. Tell me about specific examples of other successful health education campaigns in this community.

R: Yes we have so many successful programs, for example in the case of Malaria the health personnel did allots of enlightenment campaign on malaria and cholera in which they achieve what they want to achieved, they gave us mosquito net, drugs free of charge and we have to use our own money to buy them when the free once are not available. So they are successful now.

I: What are the best community channels to distribute the product?

R: The right channels to use in distributing the MNP is the village head with the help of health personnel that are working in our community, because the village head knows everybody in his community and we all know that he is honest and troth. So whatever you gave out to people, I assured you in as far as the village head concern the product will reach where you want the product to reach or intended people for the product.

I: Thanks sir. I want to hear some specific messages you think would effectively promote the product.

R: I have told you earlier that the village head with the help of health personnel that are working in our community and town announcers.

I: Please could I hear some specific message you think would effectively promote the MNP product?

R: The message should be detail explanation on how it should be used and how important it is for the children.

I: Ok. What other specific messages could be used to ensure that it will be used appropriately?

R: Enlightenment campaigns, educating our people on how the MNP product should be given; how it should be use; its importance and positive effect on the children. If possible use our indigenous people to be explaining these product and use of female for their female counter part. If that can be achieved I assured you that the product will reach where you are not even expecting.

I: Thank you so much for that message. What specific messages could be used to promote its use but limit the sharing of it?

R: The message should be detail explanation on how it should be used and how important it is for the children. You should explain to caregivers that these is the target children for the MNP product and is main for them alone, so that you can limit MNP sharing among caregivers or friends.

I: Thank you so much. Finally, is there anything else you can share about your experience with this product, placement, price?

R: I am ok with the product; the price too is ok for me as i said earlier that 100 Naira per sachet. There is no need to change anything from the product, so we are happy and excited about this product.

I: What else would you recommend to develop an effective programme?

R: My recommendation is that you should make the MNP product available within our own reach and if possible we want it to free of charge.

I: Thanks.
